# Applying RE-AIM to evaluations of Veterans Health Administration Enterprise-Wide Initiatives: lessons learned

**DOI:** 10.3389/frhs.2023.1209600

**Published:** 2023-07-28

**Authors:** Rachael R. Kenney, Robert P. Klocko, Chelsea E. Manheim, Ashley C. Mog, Jessica P. Young

**Affiliations:** ^1^ Seattle-Denver COIN, Veterans Health Administration, Denver, CO, United States; ^2^ VA Collaborative Evaluation Center, Veterans Health Administration, Denver, CO, United States; ^3^ Seattle-Denver COIN, Veterans Health Administration, Seattle, WA, United States; ^4^ VA Collaborative Evaluation Center, Veterans Health Administration, Seattle, WA, United States

**Keywords:** evaluation, quality improvement, Veterans Health Administration—VHA, rural health, implementation science, RE-AIM evaluation framework

## Abstract

**Introduction:**

The United States Veterans Health Administration (VHA) Office of Rural Health funds Enterprise-Wide Initiatives (system-wide initiatives) to spread promising practices to rural Veterans. The Office requires that evaluations of Enterprise-Wide Initiatives use the Reach, Effectiveness, Adoption, Implementation, and Maintenance (RE-AIM) framework. This presents a unique opportunity to understand the experience of using RE-AIM across a series of evaluations. The authors conducted a study to document the benefits and pitfalls of using RE-AIM, capture the variety of ways that the team captured the elements of RE-AIM, and develop recommendations for the future use of RE-AIM in evaluation.

**Materials and methods:**

The authors first conducted a document review to capture pre-existing information about how RE-AIM was used. They subsequently facilitated two focus groups to gather more detailed information from team members who had used RE-AIM. Finally, they used member-checking throughout the writing process to ensure accurate data representation and interpretation and to gather additional feedback.

**Results:**

Four themes emerged from the document review, focus groups, and member checking. RE-AIM: provides parameters and controls the evaluation scope, “buckets” are logical, plays well with other frameworks, and can foster collaboration or silo within a team. Challenges and attributes for each RE-AIM dimension were also described.

**Discussion:**

Overall, participants reported both strengths and challenges to using RE-AIM as an evaluation framework. The overarching theme around the challenges with RE-AIM dimensions was the importance of context. Many of these benefits and challenges of using RE-AIM may not be unique to RE-AIM and would likely occur when using any prescribed framework. The participants reported on the RE-AIM domains in a variety of ways in their evaluation reports and were not always able capture data as originally planned. Recommendations included: start with an evaluation framework (or frameworks) and revisit it throughout the evaluation, consider applying RE-AIM PRISM (Practical Robust Implementation Framework) to gain a broader perspective, and intentionally integrate quantitative and qualitative team members, regardless of the framework used.

## 1. Introduction

Almost a quarter of United States Veterans (Veterans) reside in rural areas after their military service and Veterans are overwhelmingly more likely to live rurally than the general population. Rural Veterans are also significantly more likely to enroll in the US Veterans Health Administration (VHA) for healthcare than Veterans who live in more urban areas (1).

The VHA established the Office of Rural Health (ORH) in 2006 with the goal of conducting, coordinating, promoting, and disseminating research on Veterans that live in rural communities (2). In 2016, ORH established Enterprise-Wide Initiatives (EWIs) (i.e., system-wide initiatives) as one way to make promising practices available across the United States. Programs that are selected as Enterprise-Wide Initiatives receive 3 years of funding with the goals of expanding rural care, integrating new programs/initiatives into the VHA system of care, and sustaining the programs after the initial funding ends. By Fiscal Year 2022, ORH funded over 50 Enterprise-Wide Initiatives (3).

In 2017, ORH began requiring evaluations of Enterprise-Wide Initiatives to use the Reach, Effectiveness, Adoption, Implementation, and Maintenance (RE-AIM) framework (4) to structure evaluation reports. This is one of only two identified instances where the entire RE-AIM framework was required to be used as an evaluation framework across a series of evaluations, the other being the Robert Wood Johnson Foundation's Prescriptions for Health (5).

RE-AIM is an implementation framework that considers elements of implementation (such as characteristics of sites and those that provide the intervention) and program performance (such as clinical outcomes). Additionally, the RE-AIM dimensions consider the steps necessary to successfully monitor and improve program implementation and success (4). ORH selected RE-AIM because reach captures the number of rural Veterans served, implementation supports the focus on equity across populations, and maintenance highlights the goal of sustainment in the VA enterprise (6). See Table 1 for the Definitions of RE-AIM Dimensions.

**Table 1:** Definitions of RE-AIM dimensions.

Dimension	Office of rural health definition (2)
Reach	The absolute number, proportion, and representativeness of individuals who are willing to participate in a given initiative, intervention, or program.
Effectiveness	The impact of an intervention on important outcomes, including potential negative effects, quality of life, and economic outcomes
Adoption	The absolute number, proportion and representativeness of settings and staff who actually initiate a program
Implementation	How closely did the facilities and staff adhere to the various elements of an intervention's protocol, including consistency of delivery as intended and the time and cost of the intervention
Maintenance	The extent to which a program or policy becomes institutionalized or part of the routine organizational practices and policies

While there are many papers and reports of individual evaluations that used RE-AIM as an evaluation framework (7–13), and papers that use RE-AIM to synthesize information across multiple evaluations (14–24), fewer authors have addressed the utility of RE-AIM as an evaluation framework (25). The purpose of this manuscript is to capture one evaluation center's experience of using RE-AIM as an evaluation framework across a series of Enterprise-Wide Initiative evaluations. Specifically, this paper aims to:

1. Document the benefits and pitfalls of using RE-AIM
2. Document the variety of ways that the team captured the elements of RE-AIM
3. Develop recommendations for the future use of RE-AIM in evaluation.

## 2. Materials and methods

### 2.1. Study design

The authors are Masters (JPY, RPK, RRK, CEM) and PhD (AM) educated Health Science Specialists with degrees in Public Health, Social Work, Sociology, Clinical Psychology, and Women, Gender, and Sexuality studies. They have 4–15 years of experience in evaluation and work at the Seattle-Denver Center for Innovation in Veteran-Centered and Value-Driven Care (COIN). The Seattle-Denver COIN has completed six Enterprise-Wide Initiative evaluations, with four more currently underway (Table 2). Four of the authors (RK, RRK, CEM, JPY) have 1–5 years of experience managing evaluations of Enterprise-Wide Initiatives and the fifth author (AM) has experience as a qualitative analyst for an Enterprise-Wide Initiative.

**Table 2:** Evaluations of enterprise-wide initiatives.

Years	Initiative
FY18-20	Accessing Telehealth Through Local Area Stations (ATLAS) (26)^a^ provides locations to access VA telehealth close to home for rural Veterans who cannot access telehealth to the home due to low internet bandwidth, discomfort with technology, lack of privacy, or other reasons.
FY18-present	The Medical Foster Home Program (27)^a^ is a long-term care option where Veterans receive long term care in their home from caregivers who live in their communities.
FY21-present	Mobile Prosthetic Orthotic Care (MoPOC) (28)^a^ provides orthotic and prosthetic services to patients in or near their home at a VA Community Based Outpatient Center via a mobile van.
FY21-present	Oral Telemedicine^a^ utilizes store-and-forward and Clinical Video Telehealth to connect rural Veterans to expert consultation from board certified specialists in oral pathology, oral medicine, and oral maxillofacial surgery to expedite diagnosis and treatment of infections and diseases of the head and neck.
FY18-19	Specialty Care Access Network-Extension for Community Healthcare Outcomes (SCAN-ECHO) (29) increases access to specialty care services for Veterans in rural and medically underserved areas by connecting specialty care teams with primary care providers to review cases and recommend treatment plans.
FY18-20	Simulation Learning, Evaluation, Assessment, and Research Network (SimLEARN) (30)^a^ provides curricula and best practices for VA providers to engage in simulation-based learning on a wide variety of projects.
FY18-19	State Veterans Telehealth^a^ is an initiative to connect Veterans in State Veterans Homes to VA Medical Centers for specialty care via telehealth.
FY21-present	Telediabetes (31)^a^ connects rural Veterans who have uncontrolled diabetes with endocrinologists, nurses, and other diabetes health professionals via clinical video telehealth or telehealth to the home.
FY16-21	The Transitions Nurse Program (TNP) (32) employs a Transitions Nurse based at an urban VA medical center to improve Veterans’ transitions from the urban VA facility back to their home VA or community care provider.
FY17-20	Virtual Integrated Multisite PACT teams (V-IMPACT) (33)^a^ provided virtual primary care, mental health, and pharmacy staffing support to VA outpatient clinics with staff variances. It has since rolled into the VA's Clinical Resource Hubs (29).

^a^ One of the authors worked on the project.

The authors took an approach reminiscent of collaborative autoethnography to learn from their previous experiences and inform future evaluations of Enterprise-Wide Initiatives. In collaborative ethnography, the personal experiences of the researchers are the primary data (34). This project was a partial collaborative autoethnographic approach; the authors were involved in all stages of the project, but the other team members were involved in specific activities (i.e., focus groups and member checking, Table 3).

**Table 3:** Project participation.

	Study design	Document review	Invited to focus group	Participated in focus group	Facilitated focus groups	Identification of themes	Participated in member checking^b^
Author/project manager/qualitative analyst (*N *= 4) (CEM, JY, RPK, RRK)	4	4	4	4	–	4	4
Author/qualitative analyst (*N *= 1) (AM)	1	1	–	–	1	1	–
Principal investigator^a^ (*N *= 6)	–	–	6	3	–	–	3
Project manager/qualitative analyst (*N *= 3)	–	–	2	2	–	–	3
Qualitative methodologist (*N *= 2)	–	–	2	2	–	–	1
Quantitative methodologist (*N *= 1)	–	–	1	1	–	–	–
Qualitative analyst (*N *= 3)	–	–	–	–	–	–	3
Quantitative analyst or programmer (*N *= 5)	–	–	–	–	–	–	4
Total (*N* = 25)	5	5	15	12	1	5	18

^a^ Three principal investigators who were invited to a focus group were also qualitative methodologists and a fourth was also a quantitative methodologist.

^b^ Everyone was invited to participate in member checking.

The first step was a document review to identify how each RE-AIM dimension was measured, challenges with using RE-AIM as an evaluation framework, data collection methods, project goals, outcomes of interest, and adaptations to RE-AIM.

The document review was followed by focus groups. Completing the document review first grounded the authors in how RE-AIM was used in the evaluations, which allowed them to ground the focus group questions in the existing documentation and use focus groups to explore personal experiences that were not captured in the existing documents. The aim of the focus groups was to gain a deeper understanding of experiences using RE-AIM to conduct an evaluation.

Finally, the authors also applied member checking techniques to ensure that the identified themes truly reflected the experiences of additional team members who worked on the evaluations that were surveyed.

### 2.2. Data collection

#### 2.2.1. Document review

The authors started by compiling relevant information and materials from prior and current evaluations. Documents reviewed included project regulatory documents, evaluation plans, logic models, local department presentations, and quarterly and annual reports (Appendix 1). Some projects did not have all documents available; they may have not had requirements to complete all documents or did not provide the files. The authors compiled documents for projects that they worked on because they had awareness of where additional files may have been stored.

Four of the authors (RK, RRK, CEM, JPY) completed a review (35) of all documents related to each specific project. The authors split up the projects and entered information into a matrix to collect data in a standardized format. The authors reviewed their own projects because they had a deeper understanding of the project nuances. The matrix included the five elements of RE-AIM, challenges, data collection methods, project goals, outcomes of interest, adaptations to RE-AIM (i.e., how RE-AIM was interpreted differently from the classic definition), and how use of RE-AIM changed over time (i.e., measuring different things in different years, adding new things over time, setting out to measure things that didn't work as expected).

#### 2.2.2. Focus groups

All authors collaborated on focus guide development which was informed by document review. The authors chose to conduct focus groups over individual interviews in order to allow for “depth and detail” through group discussion of experiences with using RE-AIM as an evaluation framework (36). Focus group questions asked about how RE-AIM was used, how use of RE-AIM changed from the original plans (e.g., planned outcomes that the team was unable to capture), challenges to using RE-AIM as an evaluation framework, and ways that RE-AIM was helpful (Appendix 2).

Key players from each project, including the principal investigator, project manager, qualitative methodologist, and quantitative methodologist, were invited via email to participate in one of two planned virtual focus groups. Each focus group targeted a subset of projects so teams could build on each other's comments. Author AM, who had less direct experience with evaluating Enterprise-Wide Initiatives than the other authors, facilitated two focus groups over Microsoft Teams. This allowed the remaining authors to participate in one focus group as a project team member and observe the other. A total of 15 team members (including four of the authors) were invited and 12 participated. At least one team member from each identified project participated. The focus group guide was sent to all participants in advance of the focus groups. Focus groups lasted approximately 60 min and were recorded and automatically transcribed in Microsoft Teams. Participants were informed that their participation was voluntary and that they could leave at any time.

### 2.3. Data analysis

#### 2.3.1. Identification of themes

After the focus groups, the authors reviewed the recordings and transcripts to identify themes. Each author independently listened to the focus group audio and conducted careful line-by-line reading of the transcript of one of the two focus groups to identify key concepts and meaning units (37). The authors then met to compare their results which were used to build and refine themes. Differences were resolved through discussion and consensus building.

#### 2.3.2. Member checking

The authors presented the identified themes at a standing meeting comprised of team members who evaluated Enterprise-Wide Initiatives. The purpose of this presentation was to check the validity, trustworthiness, and credibility of the analysis. All focus group invitees were invited to participate in member checking and member checking was opened up to other roles that were not invited to focus groups, such as qualitative analysts, quantitative analysts, and programmers (38, 39).

Finally, the authors shared an early version of this paper with all team members who participated in evaluations of Enterprise-Wide Initiatives. Additional feedback was solicited over email, in a team meeting, and in one additional meeting that was set up to capture the perspectives of team members who were unable to participate in other member checking activities. The themes presented below were synthesized across results of the document review, focus group analysis, and member checking.

This work was deemed quality improvement by the Eastern Colorado Research and Development Committee because it was designed and conducted for the purposes of improving internal VA processes in support of activities carried out in accordance with the VA mission and the institutional requirements.

## 3. Results

15 evaluators (including four of the authors, CEM, RPK, RRK, JPY) were invited to focus groups and 12 (including four of the authors, CEM, RPK, RRK, JPY) attended. Nine focus group participants (including four of the authors, CEM, RPK, RRK, JPY) and an additional 11 team members who were not invited to focus groups (qualitative analysts, quantitative analysts, and programmers) participated in member checking. At least one person from each evaluation participated in focus groups and member checking (Table 3).

Themes are grouped into overarching comments about RE-AIM and specific comments about measuring each element of RE-AIM.

Input from member checking is integrated into the themes and noted where substantial changes were made due to member checking. Overall, member checking strengthened the existing themes and did not identify new themes.

### 3.1. Overarching comments about RE-AIM

#### 3.1.1. RE-AIM provides parameters and controls the evaluation scope

Using RE-AIM in evaluation planning led to early identification of data collection methods and clearly defining each RE-AIM dimension as it related to the subject of the evaluation. Participants found it “really helpful” to do up front work to define RE-AIM dimensions before beginning the evaluation. They also commented that without the RE-AIM framework they could “write a 100-page report.”

“*Early on we knew that we needed to use RE-AIM… so we made a joint display that had the qual and quant methods in the table with every [dimension] of the RE-AIM framework listed… and listed our data sources or data variables that we would be using*.” (Principal Investigator/Qualitative Methodologist)

Even though RE-AIM was seen as useful to control the scope, some participants expressed that it was challenging when there was pertinent information that didn't fit clearly into a RE-AIM dimension. The Office of Rural Health provides a standardized report template and the participants reflected that this led to “stuffing,” “pushing,” or “fitting” findings into RE-AIM. Another challenge was when findings could be reported under multiple RE-AIM dimensions, specifically overlap between adoption and implementation. This made it difficult to decide where to include the information in the report.

“*And [Adoption] often overlaps so much with implementation… you're either double talking into sections or you're just trying to figure out ‘what am I gonna fit here or here’ when often it’s an interplay between implementation and rollout and barriers and facilitators. And then whatever you're calling adoption and how that actually played out and what your metrics are*.” (Project Manager)

Other participants commented that RE-AIM was broad by nature and didn't limit the evaluation: “I don't think I would want us to turn away from an outcome that was appropriate for, you know, representing the impact of a program” (Principal Investigator/Qualitative Methodologist). Another participant explained: “let [the RE-AIM dimensions] guide data collection, but don't be constrained in terms of expanding the data collection and analysis” (Principal Investigator/Qualitative Methodologist) These participants advised that this supplemental information that didn't “fit” elsewhere in RE-AIM could be reported in the implementation section.

#### 3.1.2. RE-AIM “buckets” are logical

Participants felt that RE-AIM was logical, and that any good evaluation would have data for each of the RE-AIM dimensions. They discussed “filling the RE-AIM buckets,” or making sure that there was data for each element of RE-AIM, when planning the evaluation. One participant commented that they felt that use of RE-AIM was not overly restrictive because the dimensions were so “high level” and another commented that “if you do a full summative evaluation from soup to nuts, you should have stuff in each of those categories” (Qualitative Methodologist)

“*RE-AIM is just kind of a logical construct like “OK, we need to find the patients and we need to see how the team is adopting it within the VA. All those things are logical things and evaluation is going to work on*.” (Project Manager/Qualitative Analyst)

Even though there is no explicit guidance that every RE-AIM dimension should be given the same amount of attention, participants still felt that it was important to explicitly recognize that there should not be an expectation that each RE-AIM dimension hold the same weight. They shared that the focus will naturally shift for different partners, initiatives/programs, and stages of implementation. Some partners were interested in effectiveness (i.e., clinical outcomes), while others were more interested in adoption (i.e., how to spread the initiative). Participants stated that they work closely with partners to ensure that the evaluation focuses on the most important or salient components, rather than trying to give equal weight to each element of RE-AIM: “*It tends to be that one or two of the components get the vast preponderance of the energy and effort and the other* *ones* *kind of end up being, I don’t want to go as far as, say, is vestigial. But we're approaching something like that depending upon what the stakeholder interests are*.” (Quantitative Methodologist)

#### 3.1.3. RE-AIM plays well with other frameworks

While most participants felt that RE-AIM was a logical framework, they commented that it didn't encompass everything of interest: “*We* *don’t think RE-AIM is always the best implementation science framework for the EWIs [Enterprise-Wide Initiatives] we've worked on to really show the barriers and facilitators*” (Qualitative Methodologist). Participants reported having success when pairing RE-AIM with other frameworks such as CFIR (40) (to “make sure you’re collecting the data you want”), PRISM (41) (for pre-implementation work), Stirman (42) (to track and explain adaptations), and Kirkpatrick (43) (for further understanding in effectiveness, adoption, and implementation results). These additional frameworks are utilized in a wide range of settings and are not specific to a VA setting.

#### 3.1.4. RE-AIM can foster collaboration or silo within a team

Some participants reflected that starting with RE-AIM fostered collaboration between qualitative and quantitative team members early in the evaluation process. Participants shared that RE-AIM can be an opportunity for intentional alignment around goals and methods if it is used early on and throughout the evaluation.

Other participants commented that use of RE-AIM siloed quantitative and qualitative efforts and without intentionality RE-AIM exacerbated existing silos. They commented that quantitative team members primarily focused on the measures of reach and effectiveness, while qualitative team members focused on the remaining dimensions of RE-AIM. Participants reflected that the team might define how each element of RE-AIM would be captured during the planning phase, but then not come back together again as a full team until reporting. These participants felt that when teams were not intentional, the nature of the RE-AIM dimensions led evaluations to be “siloed” and multi-methods, rather than true mixed methods.

“*The evaluation is kind of siloed where you have just quant folks focusing primarily on effectiveness and reach, whereas we have a lot of qualitative folks who kind of complete the rest of RE-AIM…. It seems like it would be more effective if it was more of a mixed methods kind of group sitting down and each of them having kind of equal weight on how to determine what measurement to use for each component of RE-AIM. But it tends to be quite the work itself is like quite siloed. It’s like, ‘OK, here, we're gonna have the analysts work on this’ and then they just bring it back to us. Instead of being kind of integrated*.” (Project Manager/Qualitative Analyst)

During member checking, a third perspective emerged. Some team members shared that they believed that the collaboration or lack thereof was due to the Principal Investigator's style and not the evaluation framework that was used. One of these team members commented that they were not aware of RE-AIM prior to participating in member checking.

### 3.2. Measuring the RE-AIM dimensions

All evaluations reported RE-AIM dimensions in ways that were specific to the program that they evaluated. Cross cutting themes regarding measuring RE-AIM were: (1) some outcomes were reported in multiple places, sometimes places that did not seem to fit the definition of the RE-AIM element, (2) some proposed outcomes were dropped because of inability to identify appropriate data sources, and (3) the participants utilized resources from the VA Office of Rural Health (3) and an article titled “Qualitative approaches to use of the RE-AIM framework: rationale and methods” (44) when designing and conducting their evaluations. These themes are summarized in Table 4.

**Table 4:** Themes by RE-AIM element.

Dimension	Theme
Reach	Reporting reach can be misleading—reach in early implementation is not representative of what reach will be when a program reaches maintenance
Effectiveness	Economic analysis may not accurately represent effectiveness for new initiatives—costs in early implementation are not representative of ongoing program costs
Adoption	Adoption requires context—knowing yes/no whether the program was adopted is not enough, it is important to include information about characteristics of those who do, and do not, adopt
Implementation	Additional/Supplementary data can often be included in implementation—data that might not seem to “fit” into RE-AIM is often related to implementation factors
Maintenance	It takes time for a program to reach maintenance—“maintenance” data in early implementation is speculative and may not represent actual maintenance

#### 3.2.1. Reach

##### 3.2.1.1. Reporting reach can be misleading

Some participants identified limitations with the application of Reach, especially in early implementation phases. They commented that the number of patients served in early implementation is not representative of the number of patients that will be served when the initiative reaches maintenance. In these cases, participants provided supporting information in their reports to provide context when reporting reach.

“*I find that Reach isn’t that helpful in the [Enterprise-Wide Initiatives] we've worked on because the programs we've been evaluating take a lot of effort to build… the reach metric is just often problematic with us for our efforts and honestly not that useful. Whereas I think other programs that can draw from like big secondary data sources or whatever might have a better, more effective use of reach*.” (Qualitative Methodologist)

The document review revealed that nearly all evaluations reported the number and proportion of eligible Veterans served and included information on representativeness. Other items included provider impressions of Veterans who do and do not participate, potential locations for future sites, the number of encounters, program utilization by service line and modality, and fidelity to key program components. One evaluation team proposed but was ultimately unable to capture the number of referrals to the program, number of patients offered the program, and the number of patients who decline the program.

#### 3.2.2. Effectiveness

##### 3.2.2.1. Economic analysis may not be an accurate representation of effectiveness for new initiatives

Some participants noted that cost may not be an appropriate metric for programs that are in early implementation. They reflected that new programs are often working to increase reach, which can lead to increased cost, and do not reflect the cost of the program once it reaches maintenance. Participants reported that one project looked at the prevention of death as an alternative to cost.

“*This notion that we're going to save money and provide more care at the same time is rarely realized in practice*.” (Principal Investigator/Quantitative Methodologist)

“*Cost isn’t an effective measure, it’s not really that meaningful because there’s just such a huge startup cost to starting these things and then trying to quantify the cost per patient and it just looks like ‘why are we doing this? It’s so expensive.’ but because it hasn’t already reached Adoption. Or Maintenance. That that number is not really representative of what the actual cost would be if this was something that’s stained*.” (Project Manager/Qualitative Analyst)

“*We were able to show that the program was expensive but–like, because the program prevented death among participants we were able to show like a cost benefit in a sense…. it’s really hard because people, I mean the people creating these programs tend to want to show a positive return on investment because they think that’s what they need in order for the program to be sustained*.” (Principal Investigator/Qualitative Methodologist)

Other measures of effectiveness that were identified in the document review included descriptions of the experience of participating in the program from both VHA and Veteran perspectives, perceived quality of care, satisfaction, acceptability, and feasibility. Some evaluations included measures of cost. Other information reported in effectiveness was specific to each program or initiative and included access to care, the frequency and values from clinically relevant lab draws, medications prescribed, adverse events, training results, and relational coordination (45). Outcomes that were proposed but were unable to be reported on included wait times, missed/canceled appointments, and changes in the number of Veterans served.

#### 3.2.3. Adoption

##### 3.2.3.1. Adoption requires context

One participant described that adoption was intended to be measured as yes/no and it was challenging to find a metric that “feels adequate” to report adoption. Participants described reporting barriers and adaptations in addition to raw numbers of sites and staff who adopted the initiative to provide context and more authentically represent adoption.

“*I think adoption is also problematic to me because if you if you bring it down to a ‘yes’ or ‘no’ and whether it’s yes or no overall or yes or no, these program elements were adopted or yes or no, you know, these sites were adopted and what does that mean? There’s, you know, it can seem punitive, or like, I can see where partners might be concerned… But there’s reasons [sic], right. And often it’s not just like, no, they didn’t care. It’s often like ‘we did something different because of XY or Z,’ But there’s not a great–it doesn’t feel like adoption to me is the right way to talk, to talk about that narrative, really*.” (Project Manager/Qualitative Analyst)

The document review revealed that while the evaluation reports and other artifacts included the number of settings that initiated the program being evaluated, they did not always include information about representativeness of staff and settings. Other information included in adoption was barriers and facilitators to site and VHA provider/staff participation, adaptations to the program, services and modalities implementing the program, equipment purchased, and staffing. This was another place where one evaluation planned but was unable to collect data on referrals to the program.

#### 3.2.4. Implementation

##### 3.2.4.1. Additional/supplementary data can often be included in implementation

Participants reported that RE-AIM is broad by nature, and that any additional or supplemental data collected would fit “under” implementation in the RE-AIM report and “if it didn't fit RE-AIM, I don't think I would want us to turn away from an outcome that was appropriate for, you know, representing the impact of a program” (Principal Investigator/Qualitative Analyst) One team member explained that even though RE-AIM may seem limiting if it is seen as the only way to design the evaluation, “let [the RE-AIM constructs] guide data collection, but don't be constrained in terms of expanding the data collection and analysis.”

“*There’s a little bit of tension between working toward RE-AIM and the best thing for the evaluation and the best thing for working with your partners. Which may have a lot of overlap or… it may diverge and not clearly fit RE-AIM*” (Project Manager/Qualitative Analyst)

The document review uncovered that teams included information about site context and fidelity to core program components as well as adaptations when reporting implementation. They also included information about the number and types of visits conducted, cost, acute care, acceptability, feasibility, and barriers and facilitators to implementation among other measures.

#### 3.2.5. Maintenance

##### 3.2.5.1. It takes time for new initiatives to reach maintenance

Participants reported that many evaluations that began the evaluation process when in pre-implementation or early-implementation did not reach maintenance before the 3-year evaluation ended and that there often was not robust maintenance data to report. Participants reported that they utilized qualitative data about what the partners thought was necessary to maintain the program and referred to this as “perceived maintenance.” The participants noted that “perceived maintenance” may not predict actual maintenance and associated barriers and facilitators. Some participants shared that another strategy they used was to include maintenance data from the first wave of a program to demonstrate what the expected maintenance might look like.

“*A lot of the projects that I've worked on don’t actually reach Maintenance, so it’s more about perceptions of Maintenance and sustainability that we are measuring there because we're not at a place where there is actual Maintenance*.” (Project Manager/Qualitative Analyst)

As most of the programs were evaluated before they reached maintenance, teams included perceptions of sustainability, suggestions for future program development, and plans for program sustainment in the maintenance section of their reports.

## 4. Discussion

The purpose of this manuscript was to capture the authors’ teams’ experiences using RE-AIM as an evaluation framework across a series of US Veterans Health Administration Enterprise-Wide Initiative evaluations.

Benefits and pitfalls to using RE-AIM as an evaluation framework (Aim 1) were identified in this manuscript. Overall, participants saw both strengths and challenges to using RE-AIM. Participants reported that RE-AIM provided parameters and controlled the evaluation scope, was logical, and paired well with other frameworks. While using RE-AIM as a framework sometimes seemed to improve team communication in some cases, this was not always true and may have more to do with the Principal Investigator than the RE-AIM framework. Other challenges included the importance of providing additional context, limitations of the RE-AIM dimensions, and challenges when RE-AIM seemed to miss important outcomes.

These challenges can be mitigated by thoughtful identification of ways to capture RE-AIM elements (Aim 2) and developing and implementing recommendations for future use of RE-AIM in evaluation (Aim 3). The document review revealed that some measures were reported in multiple RE-AIM elements, some of which may seem to be the “wrong” place, and that some measures were planned and later dropped. While there are tools available on the RE-AIM website that expand on how to measure the RE-AIM elements, the participants primarily consulted guidance from the funder to guide evaluations. This guidance was not as comprehensive as the tools available on the RE-AIM website. Additional recommendations are shared below.

The overarching theme around the challenges with RE-AIM dimensions was the importance of context. Participants reported that without implementation context, often provided by qualitative data, quantitative data about RE-AIM could misconstrue the actual viability of a program, especially in early implementation. While RE-AIM was initially created as a quantitative framework, more recent work points to the importance of including qualitative data when using RE-AIM (44). The integration of qualitative and quantitative data provides an opportunity for increased communication between quantitative and qualitative team members, which is reportedly seen as a good starting point for mixed method approaches to evaluation and is required for the triangulation of qualitative and quantitative data (46). This also provides opportunity for true mixed, rather than multi, method evaluations.

Finally, many of these benefits and challenges of using RE-AIM may not be unique to RE-AIM and would likely occur when using any prescribed framework. In order to understand benefits and challenges unique to RE-AIM, one would need to conduct a comparative analysis of evaluations that were required to use different frameworks. This work sets up the opportunity to look at the transferability of experience between models.

### 4.1. Situating this work in the literature

Several findings from this paper were previously addressed in an article written by the founders of RE-AIM (47). In this article, the founders of RE-AIM addressed the importance of context and have expanded to the RE-AIM PRISM (Practical Robust Implementation and Sustainability) model to stress the importance of context. Like the focus group participants, the RE-AIM founders also stated that it is a misconception that all RE-AIM dimensions must be used in every project, and they recognized that RE-AIM can be combined with other frameworks. The RE-AIM founders disputed the claim that RE-AIM dimensions are challenging to understand and stated that there are specific definitions and examples available. But the outcomes reported in Table 5, and the fact that the focus group participants did not mention referencing these products, suggest that there is a need for further dissemination of these resources.

**Table 5:** Ways that projects measured RE-AIM dimensions.

	Definition	Outcomes	
Reach	The absolute number, proportion and representativeness of individuals who are willing to participate in a given initiative, intervention or program	Number of patients served	Total number of encounters and average number encounters per patient^b^
		Provider impressions of Veterans who are served, are offered the program and decline, or are unenrolled due to dissatisfaction	Fidelity (e.g., are the screenings required by the program completed, provider training attendance)^b^
		Proportion of eligible patients served	*Number of referrals to the program* ^a^
		Representativeness of patients served	*Number of patients who are offered the program* ^a^
		Program utilization by service and by service modality^b^	*Number of patients who declined the program* ^a^
		Potential locations for future sites^b^	
Effectiveness	The impact of an intervention on important outcomes, including potential negative effects, quality of life, and economic outcomes	Risk Ratios (ED Visit, PQI hospitalization, AUDIT-C, PHQ-2, Influenza Vaccination)	Adverse events (e.g., PQI Hospitalizations, emergency room visits)
		Satisfaction and perceived satisfaction	Change in the number of Veterans served
		Perceived quality of Care	Frequency and outcomes from clinically relevant lab draws (e.g., A1c, lipid panel, vitamin B12)
		[for a training program] Intermediate training results	Feasibility^b^
		Cost analysis (e.g., return on investment, cost of the program compared to an alternative program, cost per site, cost per encounter)	Access to care (e.g., were the screenings required by the program completed? Perceived access from interviews)^b^
		Acceptability	Description of experience^b^
		Medications prescribed	*Missed/canceled appointments* ^a^ ^,^ ^b^
		Relational coordination of site staff	*Wait times* ^a^
Adoption	The absolute number, proportion and representativeness of settings and staff who actually initiate a program	Number of VA sites engaged	Services and modalities of care initiated
		Number of partner sites engaged	Equipment Purchased
		Number of providers engaged	Adaptations^b^
		Barriers and facilitators to site participation	*Number of referrals to the program* ^a^
		Staffing	
Implementation	How closely did the facilities and staff adhere to the various elements of an intervention's protocol, including consistency of delivery as intended and the time and cost of the intervention	Barriers and facilitators to implementation	Acceptability^b^
		Adaptations	Feasibility^b^
		Use of community care	Site context^b^
		Fidelity to core program components	Cost of services (e.g., equipment, staffing, space, travel)^b^
		Attrition from VA care	Number and types of visits conducted; average number of encounters per patient^b^
		Medication adherence	Acute care (hospitalizations, ED visits, UC visits)^b^
		VA Travel Reimbursement	No-show rates^b^
Maintenance	The extent to which a program or policy becomes institutionalized or part of the routine organizational practices and policies	Perceptions of sustainability	
		Suggestions for future program development	
		Plans for program sustainment	

^a^ Planned but unable to capture.

^b^ Reported in more than one element or could be reported in a different element.

The findings in this manuscript echo previous work, such as Kwan et al. (25) and Quinn et al. (48). Kwan and colleagues interviewed 17 representatives from programs and evaluators from a variety of programs that used RE-AIM while Quinn et al., surveyed 18 case study developers who used RE-AIM in developing case studies about clean fuel cooking programs in low resource countries. Like this manuscript, Quinn et al., also identified challenges with applying RE-AIM to programs that were not fully implemented. Additionally, both Kwan et al. and Quinn et al. found that RE-AIM was useful for planning and there were challenges differentiating RE-AIM constructs. While this manuscript found that barriers and facilitators were reported in adoption and implementation and contextual information was included throughout the reports, Quinn et al. found that case study developers felt that barriers and facilitators were difficult to fit into RE-AIM and they were unsure where to fit in contextual information.

Unlike this manuscript, Kwan et al. and Quinn et al., reported challenges with data acquisition. While this manuscript identified data acquisition issues in the document review, this was not a salient theme in the focus groups. This may be because this paper examines US Veterans Health Administration programs, and, as the largest integrated health system in the United States, there is a plethora of administrative data available. Additionally, this may not be salient in part due to the limited participation of quantitative team members, further discussed in the limitations.

### 4.2. Limitations

The biggest limitation to this project was that the authors reviewed their own work. While they took precautions, such as member checking and ensuring that all themes and examples were represented in the data, it is still likely that their own experiences played a disproportionate role in the findings. Review by those not intimately involved in the series of evaluations may identified a different set of themes.

A second limitation was that the RE-AIM framework is a requirement for evaluations of Office of Rural Health Enterprise-Wide Initiatives. This colored the experience of the participants. Focus group participants shared extensively about the requirement to use RE-AIM, the standardized reporting format, and the challenges that some faced when switching to RE-AIM after an evaluation was designed with a different framework. This detail was not included in the manuscript because it was specific to evaluations of Enterprise-Wide Initiatives. Evaluators who independently chose RE-AIM as an evaluation framework likely have different experiences using RE-AIM.

Finally, the perspectives of participants with qualitative expertise (15) far outweigh the perspectives of team members with quantitative expertise (6). This is in large part due to the limited number of team members with quantitative expertise: 8 team members with quantitative expertise and 15 team members with qualitative expertise worked on the evaluations of Enterprise-Wide Initiatives. Projects that highlight the perspectives of quantitative team members may reveal additional, and different, experiences with the RE-AIM framework.

### 4.3. Recommendations

Recommendations drawn from this analysis include:

- Start with an evaluation framework and continue to revisit the framework throughout the evaluation.
- Don't limit oneself to one evaluation framework; even if a specific framework is required, pairing it with additional frameworks may be necessary to ensure that the evaluation is comprehensive and meets the needs of the partners, other interested parties, and the evaluation funder.
- Think carefully about applying economic analysis; ensure that team members conducting economic analysis have the appropriate expertise, supplement economic analysis with contextual information, and consider conducting further research on applying economic analysis to programs that have not yet met sustainment.
- Consider RE-AIM PRISM (Practical Robust Implementation Framework) to gain a broader perspective, as the developers of RE-AIM have endorsed this expanded framework.
- Be intentional about integrating quantitative and qualitative team members; it is easy to lose sight of the possibilities for mixed methods and collaboration over the course of an evaluation.

In closing, while RE-AIM was not without its challenges, participants found RE-AIM to be a logical and practical framework for evaluating Enterprise-Wide Initiatives, especially when providing contextual information to support findings. More work is needed to understand the experience of using dissemination and implementation frameworks in evaluation. This includes further examination of the experience of quantitative team members, exploring the experiences of evaluators who chose the RE-AIM framework on their own accord, and comparing the experience of using RE-AIM with the experience of using other frameworks. This research will both provide guidance for evaluators who utilize the frameworks and shine a light on gaps and misconceptions that framework developers can address with additional guidance and/or resources.

## Data Availability

The datasets generated and/or analyzed during the current study are not publicly available due to the Department of Veterans Affairs’ regulatory compliance. Requests to access the datasets should be directed to rachael.kenney@va.gov.

## References

[1] VA.gov | Veterans Affairs ORH Rural Veterans. Office of rural health rural veterans. Available at: https://www.ruralhealth.va.gov/aboutus/ruralvets.asp (Accessed April 17, 2023).

[2] Health VO of R. VA.gov | Veterans Affairs Office of Rural Health Home. Office of rural health home. Available at: https://www.ruralhealth.va.gov/ (Accessed April 17, 2023).

[3] VA.gov | Veterans Affairs Office of Rural Health EWIs. Office of rural health EWIs. Available at: https://www.ruralhealth.va.gov/providers/Enterprise_Wide_Initiatives.asp (Accessed April 17, 2023).

[4] Glasgow RE Vogt TM Boles SM. Evaluating the public health impact of health promotion interventions: the RE-AIM framework. Am J Public Health. (1999) 89(9):1322–7. 10.2105/AJPH.89.9.1322 10474547 PMC1508772

[5] Green LA Cifuentes M Glasgow RE Stange KC. Redesigning primary care practice to incorporate health behavior change. Am J Prev Med. (2008) 35(5):S347–9. 10.1016/j.amepre.2008.08.013 18929980

[6] Matthieu M Kenney R Taylor L Garner K Golden R Sanders A Evaluation theory in the wild: the VA office of rural health and RE-AIM. Presented at: evaluation. New Orleans, LA (2022).

[7] Maw AM Morris MA Glasgow RE Barnard J Ho PM Ortiz-Lopez C Using iterative RE-AIM to enhance hospitalist adoption of lung ultrasound in the management of patients with COVID-19: an implementation pilot study. Implement Sci Commun. (2022) 3:89. 10.1186/s43058-022-00334-x 35962441 PMC9372925

[8] Grembowski D Conrad DA Naranjo D Wood S Coe NB Kwan-Gett T RE-AIM evaluation plan for Washington state innovation models project. Qual Manag Health Care. (2020) 29(2):81. 10.1097/QMH.0000000000000246 32224792

[9] Welch A Healy G Straker L Comans T O'Leary S Melloh M Process evaluation of a workplace-based health promotion and exercise cluster-randomised trial to increase productivity and reduce neck pain in office workers: a RE-AIM approach. BMC Public Health. (2020) 20:180. 10.1186/s12889-020-8208-9 32019559 PMC7001341

[10] Aoki A Mochida K Kuramata M Sadamori T Bhandari AKC Reis Freitas H The RE-AIM framework-based evaluation of the implementation of the maternal and child health handbook program in Angola: a mixed methods study. BMC Health Serv Res. (2022) 22:1071. 10.1186/s12913-022-08454-9 35996173 PMC9395902

[11] Jones LK Strande NT Calvo EM Chen J Rodriguez G McCormick CZ A RE-AIM framework analysis of DNA-based population screening: using implementation science to translate research into practice in a healthcare system. Front Genet. (2022) 13:883073. 10.3389/fgene.2022.883073 35692820 PMC9174580

[12] Verhey R Chitiyo C Mboweni S Turner S Murombo G Healey A Using the RE-AIM framework to evaluate the implementation of scaling-up the friendship bench in Zimbabwe – a quantitative observational study. BMC Health Serv Res. (2022) 22:1392. 10.1186/s12913-022-08767-9 36419089 PMC9682765

[13] Lewis MA Wagner LK Rosas LG Lv N Venditti EM Steinman LE Using RE-AIM to examine the potential public health impact of an integrated collaborative care intervention for weight and depression management in primary care: results from the RAINBOW trial. PLoS One. (2021) 16(3):e0248339. 10.1371/journal.pone.0248339 33705465 PMC7951877

[14] Brown M Roman NV. Using the RE-AIM framework to identify and describe nutritional feeding styles and intervention model best practices for primary caregivers in Africa: a narrative review. Nutr Health. (2021) 27(2):171–80. 10.1177/0260106020977053 33269666

[15] Iwelunmor J Nwaozuru U Obiezu-Umeh C Uzoaru F Ehiri J Curley J Is it time to RE-AIM? A systematic review of economic empowerment as HIV prevention intervention for adolescent girls and young women in sub-saharan Africa using the RE-AIM framework. Implement Sci Commun. (2020) 1:53. 10.1186/s43058-020-00042-4 32885209 PMC7427963

[16] Pereira EL Estabrooks PA Arjona A Cotton-Curtis W Lin JCP Saetermoe CL A systematic literature review of breastfeeding interventions among black populations using the RE-AIM framework. Int Breastfeed J. (2022) 17:86. 10.1186/s13006-022-00527-z 36528606 PMC9758845

[17] Antikainen I Ellis R. A RE-AIM evaluation of theory-based physical activity interventions. J Sport Exerc Psychol. (2011) 33(2):198–214. 10.1123/jsep.33.2.198 21558580

[18] Yang W Liang X Sit CHP. Physical activity and mental health in children and adolescents with intellectual disabilities: a meta-analysis using the RE-AIM framework. Int J Behav Nutr Phys Act. (2022) 19:80. 10.1186/s12966-022-01312-1 35799257 PMC9261031

[19] Pinheiro-Carozzo NP Murta SG Vinha LGDA da Silva IM Fontaine AMGV. Beyond effectiveness of the strengthening families program (10-14): a scoping RE-AIM-based review. Psicol Reflex Crit. (2021) 34:16. 10.1186/s41155-021-00182-z 34131838 PMC8206301

[20] Compernolle S De Cocker K Lakerveld J Mackenbach JD Nijpels G Oppert J-M A RE-AIM evaluation of evidence-based multi-level interventions to improve obesity-related behaviours in adults: a systematic review (the SPOTLIGHT project). Int J Behav Nutr Phys Act. (2014) 11:147. 10.1186/s12966-014-0147-3 25480391 PMC4266878

[21] Linder S Ferschl S Abu-Omar K Ziemainz H Reimers AK. Evaluating physical activity interventions for socioeconomically disadvantaged adults through the RE-AIM framework: a systematic review of experimental and non–/quasi-experimental trials. Prev Med Rep. (2022) 29:101943. 10.1016/j.pmedr.2022.101943 36161121 PMC9502049

[22] Nwaozuru U Obiezu-Umeh C Obi-Jeff C Shato T Gbaja-Biamila T Oladele D A systematic review of randomized control trials of HPV self-collection studies among women in sub-saharan Africa using the RE-AIM framework. Implement Sci Commun. (2021) 2:138. 10.1186/s43058-021-00243-5 34911573 PMC8672475

[23] Asare M Popelsky B Akowuah E Lanning BA Montealegre JR. Internal and external validity of social media and mobile technology-driven HPV vaccination interventions: systematic review using the reach, effectiveness, adoption, implementation, maintenance (RE-AIM) framework. Vaccines. (2021) 9(3):197. 10.3390/vaccines9030197 33652809 PMC7996801

[24] Haire-Joshu D Morshed AB Phad A Johnston S Tabak RG. Applying RE-AIM to evaluate the external validity of weight gain prevention interventions in young adults: a systematic review. J Public Health Manag Pract. (2021) 27(2):154–65. 10.1097/PHH.0000000000001159 32332487 PMC7837750

[25] Kwan BM McGinnes HL Ory MG Estabrooks PA Waxmonsky JA Glasgow RE. RE-AIM in the real world: use of the RE-AIM framework for program planning and evaluation in clinical and community settings. Front Public Health. (2019) 7:345. 10.3389/fpubh.2019.00345 31824911 PMC6883916

[26] ATLAS | connected care. Available at: https://connectedcare.va.gov/partners/atlas (Accessed April 17, 2023).

[27] Haverhals LM Manheim CE Katz M Levy CR. Caring for homebound veterans during COVID-19 in the U.S. department of veterans affairs medical foster home program. Geriatrics. (2022) 7(3):66. 10.3390/geriatrics7030066 35735771 PMC9223204

[28] Diffusion marketplace. Available at: https://marketplace.va.gov/innovations/mobile-ops (Accessed April 17, 2023).

[29] VA.gov | Veterans affairs patient care services. Available at: https://www.patientcare.va.gov/primarycare/CRH.asp (Accessed April 17, 2023).

[30] VA-office of healthcare innovation and learning. Available at: https://www.innovation.va.gov/simlearn/ (Accessed April 17, 2023).

[31] Direct care TeleDiabetes practice in the Phoenix VA healthcare system: innovation report - ClinicalKey. Available at: https://www.clinicalkey.com/#!/content/playContent/1-s2.0-S1751991822000821?returnurl=https:%2F%2Flinkinghub.elsevier.com%2Fretrieve%2Fpii%2FS1751991822000821%3Fshowall%3Dtrue&referrer= (Accessed April 19, 2023). 10.1016/j.pcd.2022.04.005 35534420

[32] Gilmartin HM Warsavage T Hines A Leonard C Kelley L Wills A Effectiveness of the rural transitions nurse program for veterans: a multicenter implementation study. J Hosp Med. (2022) 17(3):149–157. 10.1002/jhm.12802 35504490

[33] Mog AC Moldestad M Kenney R Stevenson L Lee M Ho PM “I was unsure at first”: a qualitative evaluation of patient perceptions of VA clinical video telehealth visits in the V-IMPACT program. J Patient Exp. (2022) 9:23743735221107236. 10.1177/23743735221107237 PMC9260561 35813242

[34] Chang H Ngunjiri F Hernandez KA. Collaborative autoethnography. 1st ed. Walnut Creek, CA: Routledge (2013).

[35] Bowen GA. Document analysis as a qualitative research method. Qual Res J. (2009) 9(2):27–40. 10.3316/QRJ0902027

[36] Nyumba T Wilson K Derrick C Mukherjee N. The use of focus group discussion methodology: insights from two decades of application in conservation. Methods Ecol Evol. (2018) 9(1):20–32. 10.1111/2041-210X.12860

[37] Vaismoradi M Jones J Turunen H Snelgrove S. Theme development in qualitative content analysis and thematic analysis. J Nurs Educ Pract. (2016) 6(5):100. 10.5430/jnep.v6n5p100

[38] Creswell JW Miller DL. Determining validity in qualitative inquiry. Theory Pract. (2000) 39(3):124–30. 10.1207/s15430421tip3903_2

[39] Birt L Scott S Cavers D Campbell C Walter F. Member checking: a tool to enhance trustworthiness or merely a nod to validation? Qual Health Res. (2016) 26(13):1802–11. 10.1177/1049732316654870 27340178

[40] Damschroder LJ Aron DC Keith RE Kirsh SR Alexander JA Lowery JC. Fostering implementation of health services research findings into practice: a consolidated framework for advancing implementation science. Implement Sci. (2009) 4:50. 10.1186/1748-5908-4-50 19664226 PMC2736161

[41] Feldstein AC Practical GRA. Robust implementation and sustainability model (PRISM) for integrating research findings into practice. Jt Comm J Qual Patient Saf. (2008) 34(4):228–43. 10.1016/S1553-7250(08)34030-6 18468362

[42] Stirman SW Miller CJ Toder K Calloway A. Development of a framework and coding system for modifications and adaptations of evidence-based interventions. Implement Sci. (2013) 8(1):65. 10.1186/1748-5908-8-65 23758995 PMC3686699

[43] Chellaiyan V Suliankatchi R. Health research methodology workshop: evaluation with the Kirkpatrick model. Natl Med J India. (2019) 32(2):100. 10.4103/0970-258X.275352 31939408

[44] Holtrop JS Rabin BA Glasgow RE. Qualitative approaches to use of the RE-AIM framework: rationale and methods. BMC Health Serv Res. (2018) 18(1):177. 10.1186/s12913-018-2938-8 29534729 PMC5851243

[45] Bolton R Logan C Gittell JH. Revisiting relational coordination: a systematic review. J Appl Behav Sci. (2021) 57(3):290–322. 10.1177/0021886321991597

[46] Guetterman TC Fetters MD Creswell JW. Integrating quantitative and qualitative results in health science mixed methods research through joint displays. Ann Fam Med. (2015) 13(6):554–61. 10.1370/afm.1865 26553895 PMC4639381

[47] Holtrop JS Estabrooks PA Gaglio B Harden SM Kessler RS King DK Understanding and applying the RE-AIM framework: clarifications and resources. J Clin Transl Sci. (2021) 5(1):e126. 10.1017/cts.2021.789 34367671 PMC8327549

[48] Quinn AK Neta G Sturke R Olopade CO Pollard SL Sherr K Adapting and operationalizing the RE-AIM framework for implementation science in environmental health: clean fuel cooking programs in low resource countries. Front Public Health. (2019) 7:389. 10.3389/fpubh.2019.00389 31921753 PMC6932973

