# Supplementary material for: Applying RE-AIM to evaluations of Veterans Health Administration Enterprise-Wide Initiatives: lessons learned
Source: Front Health Serv. 2023 Jul 28;3:1209600. doi: 10.3389/frhs.2023.1209600 (PMC10421720; doi:10.3389/frhs.2023.1209600)
Supplement: Supplementary file 1 [file Datasheet1.zip › Data Sheet 1/Appendix1_Documents Reviewed.pdf]

Appendix 1: Documents Reviewed

**Project**	**Evaluation Plan**	**Logic Model** (Beginning in 2020)	**ORH Report**	**Local Regulatory Documents**	**Local Dept. Presentations**
**ATLAS**	X	X	X	X	X
**Medical Foster Homes**		X	X		
**MoPOC**	X	X	X	X	X
**Oral TeleMed**	X	X	X	X	X
**SCAN-ECHO**					X
**SimLEARN**	X		X		X
**State Vets Tele-Health**			X		
**TeleDiabetes**	X	X	X	X	X
**TNP**	X		X		
**V-IMPACT**	X		X	X	X
