# Supplementary material for: Applying RE-AIM to evaluations of Veterans Health Administration Enterprise-Wide Initiatives: lessons learned
Source: Front Health Serv. 2023 Jul 28;3:1209600. doi: 10.3389/frhs.2023.1209600 (PMC10421720; doi:10.3389/frhs.2023.1209600)
Supplement: Supplementary file 1 [file Datasheet1.zip › Data Sheet 1/Appendix2_FocusGroupGuide.pdf]

Appendix 2: Focus Group Guide

**RE-AIM and Enterprise-Wide Initiatives focus group guide**

Thank you for taking the time to meet with us and talk about how you use RE-AIM in your work evaluating Enterprise-Wide Initiatives or EWIs. This discussion is meant to focus specifically on how we use RE-AIM in evaluation.

The information you share will be used to compile best practices and develop a paper and about how the Seattle Denver COIN uses RE-AIM in our work.

We value your experiences and opinions and want to hear a wide range of opinions on the questions we'll be asking, and we want to be sure that everyone has a chance to voice their opinions. Please feel free to unmute and share your perspective during the conversation. We also encourage you to use the chat to share your thoughts.

No names will be associated with quotes in the paper but we would like to acknowledge your participation in the acknowledgements. Please let Rachael know if you prefer not to be named. We will circulate a draft of the paper and materials before they are shared beyond the COIN and you will have an opportunity to provide additional comments.

Here is the accepted abstract (although, this needs to be modified because there are two non-VACE EWIs that we are including):

To make sure I don't miss anything and get the full benefit of our time today, I would like to audio record this call and save the chat. The audio-file for the recording and a copy of the chat will be stored directly to a restricted access folder on the VA secure computer system. Is this okay with you all? [Hit record button.] Okay, to confirm, I'm starting the recording. Is this ok with you all?

- How did you use RE-AIM in initial planning of evaluations?
    - What came first, the evaluation questions or RE-AIM?
    - How did your team incorporate other things you wanted to measure into RE-AIM?
    - Did you use any other evaluation frameworks in addition to RE-AIM?
- Here is how we saw RE-AIM modified. Do you have other examples? *Keep discussion of modifications related to RE-AIM.*
    - *SimLEARN: When VACE took over the evaluation, existing data was not organized for any framework*
    - *Medical Foster Homes: Early in the evaluation, captured too much information, became more focused on the RE-AIM framework over time*
    - *ATLAS: When the project was not implemented on the expected timeline, shifted focus to pre-implementation*

- Here is how we saw evaluation plans change in relation to measuring RE-AIM, do you have any other examples? *Keep discussion on changes related to RE-AIM.*
    - *Telediabetes: Unable to access data about screenings completed for effectiveness measure*
    - *ATLAS: Were going to look at mental health metrics for effectiveness but ATLAS can be any type of appointment, not just Mental Health, so we did not move forward with exploring these metrics specifically. Didn't have a great way to measure effectiveness.*
- Here are some of the challenges to RE-AIM we identified, do you have other examples?
    - *Making sure everyone on the team understands the framework and differentiation between the categories.*
    - *RE-AIM doesn't capture everything. (e.g., SimLEARN adding the Kirkpatrick model to capture additional elements)*
    - PROBE: How did RE-AIM limit your evaluation?
- How was RE-AIM helpful in planning and carrying out your evaluation?
    - Was RE-AIM an appropriate framework for your evaluation?
    - In what ways is RE-AIM a useful framework?
    - What value was added from using RE-AIM?
    - What resources do you find helpful when using RE-AIM as an evaluation framework?
- What should other evaluators know about using RE-AIM as an evaluation framework?
- That brings us to the end of our questions. Is there anything else you would add about using RE-AIM as a framework to evaluate EWIs?
